# Open questions: seeking a holistic approach for mitochondrial research

**DOI:** 10.1186/s12915-015-0120-x

**Published:** 2015-02-05

**Authors:** Heidi M McBride

**Affiliations:** Montreal Neurological Institute, McGill University, 3801 University Avenue, Rm 622C H3A 2B4, Montreal, QC H3A 0G4 Canada

## Abstract

In addition to their role as energy generators, mitochondria play critical and active roles in diverse signalling pathways, from immunity to cell survival and cell fate decisions. However, there remain many open questions and challenges as we work towards integrating this mighty organelle into established paradigms of cellular physiology.

## Comment

Mitochondria are complicated. One of the most interesting things that struck me in the 20 years that I have been studying the cell biology of mitochondria is the compartmentalization of both the organelle and the field. Historically there were those who studied mitochondrial protein import or mitochondrial genetics. Others worked on bioenergetics or metabolism, and still others focused on mitochondrial dynamics, or calcium flux, cell death, and so on. It is amazing that we could focus so intently on isolated functions within this complex organelle. In fairness, to consider the entire organelle is overwhelming, and a truly integrated view of mitochondria in their native cellular environment is not an easy target. But we have now come to a moment in history where these islands of understanding must unite. I offer a few suggestions to those both old and new in the field: humble thoughts for a way forward.

## Collaborate

Most of us are not geniuses, and cannot operate with an encyclopaedic knowledge of metabolism, calcium homeostasis, tissue physiology, bioenergetics and lipid chemistry. On the other hand, clinician scientists or physiologists who hope to incorporate mitochondria into their signalling paradigms feel overwhelmed with the complexity of the organelle, the experimental approaches, and sometimes, the dogma common to such established fields. The first step is an obvious one - forge meaningful collaborations that will truly push the field forward. I think we are finally past the point where non-mitochondrial scientists simply write us off, assuming that the mitochondria are a known entity, uninteresting, boring. Indeed the potential for fundamental new concepts in mitochondrial function has never been higher, and the disease relevance is clear. Mitochondrial pathways are practically untouched as a therapeutic target, for example.

For those of us working on the fundamental aspects of mitochondrial function, we must work harder to consider the physiology of real tissues. I’m not suggesting we abandon our fundamental projects, certainly not! Basic discoveries will remain the lifeblood of clinical development. But with collaborations we can extend our studies simultaneously and move into ‘real’ cells. Adapting these models will more rapidly push our discoveries up the ladder of biomedical translation. My own collaborations have provided me with confidence and helped me to understand complex physiologies that would otherwise have not crossed my radar screen. It sounds obvious, but funding agencies and promotion mechanisms do not always reward collaborations enough. Grants need a single principal applicant and team grants can be more political than functional. It is also clear that collaborations are more difficult than simply continuing along a successful, independent track. However, understanding the complexities of mitochondrial function and signalling will require open, and sometimes challenging, collaborations.

## Embrace the unknown

Another thing that I am forced to learn over and over is that much of what I thought was true, is not. The idea that the mitochondria are simple, isolated organelles was broken wide open through a number of unexpected discoveries over the past decade. In my area of mitochondrial dynamics, it has been made clear that they operate as an interconnected reticulum, the implications of which are still being uncovered [1]. In addition, mitochondria were not supposed to participate in vesicular transport routes, something we have shown is simply not true [2]. In the signalling arena, mtDNA release can activate inflammation, and very recent studies have shown that mtDNA is released during Bax/Bak-dependent apoptosis, where it activates the cytosolic DNA-sensing machinery to transcribe inflammatory cytokines. Importantly, this pathway is blocked by apoptotic caspases, ensuring that the clean forms of cell death do not launch unnecessary inflammation [3,4]. This is yet another unexpected pathway directly linking mitochondria to immunity [5,6], alerting us to the importance of mtDNA as a signalling molecule.

Even the metabolic decisions to burn sugar versus fat were thought to be passive, based on some notion of cellular ‘need’. But these are highly tuned and disease-relevant decisions, and it turns out that the molecular mechanisms of metabolic switching underpin a fundamental aspect of cell fate decisions [7,8]. We have also learned that metabolites shuttle directly between organelles through regulated contact sites. Indeed, contacts with the endoplasmic reticulum, lysosomes/vacuoles, peroxisomes, and endosomes are hot topics of study at the moment [9-15], and I don’t imagine it will end there. I have always been sceptical of the idea that free diffusion is a major determinant of metabolic control, and these emerging studies simply reinforce my scepticism. Cell biology strikes again, let’s say. Imagine how dynamic these mitochondrial contacts must be in the nerve terminal, or how important they would be in the liver, or muscle. Exciting times lie ahead. To me, we have discovered only the very tip of what will be a massive iceberg in terms of the regulation and complexity of mitochondrial function.

## Love the ‘omics’ and assume nothing

Lastly, as we struggle for this elusive, holistic approach to mitochondrial function *in vivo*, our experimental tools must also evolve. This is certainly happening in every institution and for every field. But I submit that mitochondrial studies are particularly demanding, as they are not just carriers, or membranes that shuttle things in or out of the cell. They are living, breathing organelles making lipids, ribosomes, RNA, protein, steroids, iron-sulphur clusters, not to mention energy. In this context, we must employ an unbiased network of approaches and assume nothing. For example, the mechanisms of lipid flux between the metabolic triad of endoplasmic reticulum, mitochondria and peroxisomes are almost completely unknown in many areas, from bile acid synthesis to plasmalogen production in myelination [16]. To fully evaluate our experiments it becomes increasingly important to move beyond any anticipated output and look at the network changes. Lipidomics, metabolomics, proteomics, and transcriptomics are essential tools for this multifunctional organelle. Sadly, these techniques are often expensive and of limited availability to some, so we need to use these arguments to push the institutions forward. I am convinced that there can be no assumptions as we seek to uncover the myriad ways mitochondria are functionally integrated within the cell. Major technological advances permit an unbiased, rapid and powerful interrogation of the cellular state. There is no future in the singular approach to mitochondrial function. To me this is both the most wonderful and challenging thing facing us for the next decade.
